# Ultra low concentration deltamethrin loaded patch development and evaluation of its repellency against dengue vector *Aedes (S) albopictus*

**DOI:** 10.1186/1756-3305-6-284

**Published:** 2013-09-28

**Authors:** Pronobesh Chattopadhyay, Sunil Dhiman, Kangujam Adiya Devi, Subham Banerjee, Bipul Rabha, Ashwani Chaurasia, Vijay Veer

**Affiliations:** 1 Division of Pharmaceutical Technology, Defence Research Laboratory, Tezpur, Assam 784 001, India; 2 Division of Medical Entomology, Defence Research Laboratory, Tezpur, Assam 784 001, India; 3 Department of Pharmaceutics, Girijananda Chowdhury Institute of Pharmaceutical Science, Guwahati, Assam 781017, India

**Keywords:** Deltamethrin, Mosquito repellent, Patch, HPLC, *Aedes* (*Stegomyia*) *albopictus*

## Abstract

**Background:**

Mosquito repellents and emanators confer protection against mosquito bites through spatial action of emanated vapours which are released into the adjoining environment. Synthetic insecticides released into the environment in ultra low volume vapour phase deter the mosquitoes from biting humans in a protected space.

**Methods:**

Formulation patches were prepared using the solvent evaporation method over a backing membrane and using Dibutylphthalate (DBT) as a plasticizer. The effect of formulation variables on the deltamethrin release from the patch matrices were studied under accelerated conditions, whereas, HPLC was used for quantitative estimation of deltamethrin. The prepared patch formulations were subjected to physicochemical studies, such as, deltamethrin content, thickness, weight variation, percent moisture content, moisture uptake, surface area and surface pH determination. Deltamethrin-polymer interaction and compatibility was ascertained using DSC and FT-IR, while surface morphology and deltamethrin distribution in the patch were studied using SEM technique. Repellent activity of the patch formulations was evaluated against *Ae. albopictus* mosquitoes.

**Results:**

Blends of polymeric combinations of polyvinylpyrrolidone (PVP) and ethyl-cellulose (EC) with admixture of deltamethrin provided prolonged repellent activity against *Ae. albopictus* mosquitoes. Physicochemical characterisation indicated the suitability of deltamethrin patch formulation with the polymeric combinations of PVP and EC. Patches were very effective against laboratory reared *Ae. albopictus* mosquitoes. No significant difference was observed between the performance of test patches and commercially available repellent cream Mosqshield®.

**Conclusion:**

Deltamethrin loaded patches provided effective repellency against *Ae. albopictus* mosquitoes. The study emphasised that deltamethrin released to the environment in low concentration could be an excellent spatial repellent against hematophagous mosquitoes.

## Background

More than two billion people, mostly in tropical countries, are at risk of mosquito-borne diseases, such as malaria, dengue, Japanese encephalitis and filariasis [1,2]. *Aedes* (*Stegomyia*) *albopictus* (Diptera: Culicidae) is an epidemiologically important mosquito responsible for the transmission of many viral pathogens [2,3]. *Ae. albopictus* received considerable attention in India after recent reports indicating its potential role in disease transmission in various parts of the country [2,4-6]. Use of repellents as personal protection measures against the insect bites is the most accepted method to control insect vector borne diseases currently [7-9]. Many of the mosquito repellents, though available in the market and advertised to be effective repellents, are unpleasant and ineffective in repelling the mosquitoes [10]. However, an ideal mosquito repellent must provide a long-term protection and be effective against all mosquito species. The ideal repellent compound would prevent bites from a broad range of insect species, remain effective for a comparatively longer time, cause no irritation to skin or mucous membranes, possess no systemic toxicity, be totally greaseless and odourless and safe to all age groups including infants [11,12]. Repellents at a very low-dose may provide specific and low-toxicity augmentation to the conventional pesticides applied around houses and workplaces [13]. The most common mosquito repellent formulations available in the market containing DEET (N, N-diethyl-3-methylbenzamide), DEPA and deltamethrin have shown excellent repellency against mosquitoes and other biting insects [8,14-17]. Deltamethrin, first synthesized in 1974 and marketed in 1977, is considered to be a relatively safe synthetic mosquito repellent and widely used in tropical countries [18,19]. Deltamethrin is a synthetic pyrethroid insecticide which possesses an extremely high level of activity against a wide range of insects acting both by direct contact and ingestion [20]. Various synthetic chemicals used at toxic levels are currently the only confirmed effective strategy for mosquito vector control, but emerging insecticide resistance issues are threatening this approach [21]. Application of ultra low volume (ULV) insecticides has proved effective in managing high densities of adult vector mosquitoes [22].

The present study was designed to develop a suitable matrix type mosquito repellent patch containing deltamethrin using different blends of polymeric combinations for prolonged release of repellent against mosquitoes. This study presents a systematic approach of using deltamethrin in ultra low concentration as an effective repellent against *Ae. albopictus* mosquitoes and emphasises that use of ULV deltamethrin released into the environment could be an alternative method for controlling mosquitoes and other vector insects.

## Methods

Deltamethrin was obtained as a gift sample from Tagros India Ltd. Mumbai, India. Polyvinylpyrrolidone (PVP K-30), Dibutylphthalate (DBT), and Ethylcellulose (EC, ethoxy content 48-49.5% w/w) were obtained from Himedia Laboratories Pvt. Ltd. Mumbai, India. Chloroform and water (for HPLC) were obtained from Spectrochem Pvt. Ltd. Mumbai, India. Acetonitrile (for HPLC) was purchased from Merck Specialities Pvt. Ltd. Mumbai, India. Dimethylsulfoxide (DMSO) was purchased from Loba Cheime Pvt. Ltd. Mumbai, India. All reagents and solvents used were of analytical grade and used as received without any further purification.

### Preparation of deltamethrin loaded patches

Deltamethrin loaded mosquito repellent patches were prepared by solvent evaporation method using varying ratios of different blends of polymers. DBT was used as plasticizer at a fixed concentration of 20% w/w of dry weight of polymer. Initially, the polymers at a varied ratio were dissolved in chloroform and then deltamethrin and plasticizer were added to it. This mixture was moulded into rings with defined surface area and thickness over the backing membrane on a horizontal surface followed by solvent evaporation at an ambient temperature. The rate of evaporation was controlled by inverting the funnel. The patches formed were separated from the rings for further processing.

### Physicochemical characterization of deltamethrin loaded patches

#### Physical appearance

All the deltamethrin loaded patches were visually inspected for shape, smoothness, stickiness, clarity, homogeneity, flexibility and uniformity.

#### Uniformity of weight

Weight was determined by individually weighing three randomly selected patches from each batch of formulation. In the experiment, attention was given so that the individual weight was uniform as compared to the average weight. The weight was expressed by mean and standard deviation.

#### Surface area

Surface area of patches was studied by using a millimeter scale and expressed by mean and standard deviation.

#### Surface pH determination

The patches were allowed to remain in contact with chloroform for 2 h at room temperature, and pH was determined using pH paper.

#### Percent moisture uptake

Weighed patches were kept in a dessicator at room temperature for 48 h. These were exposed to 75.5% relative humidity over a saturated solution of aluminium chloride in a dessicator until a constant weight is achieved. The percent moisture uptake was calculated using the following formula;

$$\begin{array}{ll} \%\text{Moisture uptake}= & \text{Final weight}-\text{Initial weight}\\ & \times 100/\mathrm{Initial weight} \end{array}$$

#### Percent moisture content

Three patches from each formulation were weighed and kept in dessicators containing fused calcium chloride at 37°C until the notable weight change was observed. This weight was noted as the final weight. The percent moisture content is calculated using following formula;

$$\begin{array}{ll} \%\mathrm{Moisture content}= & \mathrm{Initial weight}-\mathrm{Final weight}\\ & \times 100/\mathrm{Final weight} \end{array}$$

#### Flatness

A patch should possess a smooth surface and should not constrict with time. This can be demonstrated in a flatness study. For flatness determination, one patch is cut from the centre (2×1 cm) and applied on the skin. The length of patch is measured and variation in length is measured by determining percent constriction. It is assumed that zero percent constriction refers to 100 percent flatness.

$$\%\mathrm{Constriction}=I_{1}-I_{2}\times 100/I_{1}$$

Where, I_2_ = Final length of each strip, I_1_ = Initial length of each strip.

#### Measurement of thickness

The thickness of the patch was measured using a digital micrometer (Mitotousu, Tokyo, Japan) at three different points of each patch and was expressed in mean and standard deviation.

### Scanning electron microscopy (SEM)

The surface morphology of the blank and deltamethrin loaded patches was studied using scanning electron microscopy (JEOL, JSM-6390 LV, England). The samples were mounted onto stubs using double sided adhesive tape and sputter coated with gold palladium. The coated patches were observed and photographed at the required magnification at room temperature.

### Estimation of deltamethrin by HPLC

The estimation of the deltamethrin was performed using HPLC (Analytical technologies limited, Gujarat, India) with a UV/visible detector and C_18_ column (Chromosil, particle size 5 μm, 250 mm × 4.6 mm). Deltamethrin was separated by isocratic elution technique with a mixture of mobile phase containing acetonitrile: water (80: 20,% v/v) at a flow rate of 1.0 ml/min and UV detection at 245 nm.

### Deltamethrin content

The patch of specified diameter was extracted with chloroform and kept for about 2 h at room temperature in order to extract the deltamethrin completely from the polymeric matrix and centrifuged at 3000 × *g* for 15 min. A fixed amount of resulting supernatant solution from above was analyzed for deltamethrin content using HPLC (Analytical technologies limited, Gujarat, India) as method described elsewhere [23].

### FT-IR spectrophotometry

The infrared data are helpful to confirm the identity of the component and to detect the interaction of the components with the polymer. Infrared spectra of deltamethrin and polymer, alone and in physical mixtures were obtained and investigated for any possible interaction between polymer and deltamethrin by FT-IR spectrophotometer (Bruker, α Alpha-E, Germany).

### Differential scanning calorimetry (DSC)

The physicochemical compatibility between the components and polymer used in the formulations of deltamethrin-loaded mosquito repellent patches were evaluated by DSC analysis. The DSC thermograms (Perkin Elmer, Jade DSC, USA) obtained for pure deltamethrin, pure polymers, their physical mixtures and formulated patches were compared to ascertain the interactions. Samples were heated at a temperature range between 50-250°C at a heating rate of 10°C/min.

### Release study

The release study was carried out under accelerated conditions of higher temperature. Patches were kept in an oven at 40°C and withdrawn at different time intervals from each batch and then extracted with chloroform and kept for about 2 h at room temperature. The solution was centrifuged at 3000 rpm for 15 min to remove the polymeric remnants and the supernatant liquid was collected and analyzed using HPLC.

### Mathematical modelling of release kinetics

To understand the mechanism of deltamethrin permeation kinetics from the developed patches, the release data were fitted to various release kinetic equations;

*Zero-order equation* (cumulative percentage drug permeated vs. time) [24]

(1) $$Q_{t}=Q_{0}+k_{0t}$$

Where, Q_t_ is the amount of deltamethrin release in time *t*, *Q*_*0*_ is the initial amount of deltamethrin in the solution (most times, *Q*_*0*_ = 0) and *k*_*0*_ is the zero order release rate.

*First-order equation* (log cumulative percentage drug remaining to be permeated vs. time)

(2) $$\ln\;Q_{t}=\ln\;Q_{0}+k_{1}t$$

Where, *Q*_*t*_ is the amount of deltamethrin released in time *t*, *Q*_*0*_ is the initial amount of deltamethrin in the solution and k_1_ is the first order release rate constant.

Higuchi’s model equation

(3) $$Q=k_{H}t^{1/2}$$

Where, Q is the amount of deltamethrin release at time *t*, and *k*_*H*_ is the Higuchi diffusion rate constant.

*Korsmeyer-peppas model*[25]

(4) $$\mathrm{Mt}/\mathrm{M\alpha}\;Kt^{n}$$

Where, *Mt* is the amount of deltamethrin released at time *t*, *M*α is the amount of deltamethrin released after infinite time, *K* is a kinetic constant incorporating structural and geometric characteristics of the formulation and *n* is the diffusional exponent indicative of the drug release mechanism.

### Mosquitoes and repellency test

*Ae. albopictus* mosquitoes are regularly maintained in the laboratory under controlled temperature (28 ± 2°C) and relative humidity (75-80%). For the present study, about 80-100 adult (3 days old) female *Ae. albopictus* mosquitoes were introduced into the customised repellent trial chamber (46 × 37 × 36 cm) through the hole on top with the help of sucking tube. Prior to testing, the mosquitoes were starved by providing them with only water for 12 h. Both hands of the test volunteers were used for testing the repellent activity. The Right hand applied with a blank patch was the placebo control, while the left hand applied with a deltamethrin loaded patch was taken as the test. The repellent activity was evaluated by inserting the hand into the test chamber for one minute at the start of the trial (0 min), after 30 min interval for the first hour of application and after an interval of 60 min for the rest of testing duration. Therefore, mosquito landing and biting was recorded eleven times during each trial. The hand was placed inside the repellent chamber through a hole up to wrist and plugged with cotton to prevent escape of mosquitoes. The test chamber had clear glass sides and front (for viewing) and a sheet aluminium bottom. Sucrose solution (10%) was available to the mosquitoes at all the times during the trial. Repellency was evaluated up to 9 h and the test mosquitoes were replaced with a new set of mosquitoes after 4 h of the trial. Commercially available DEPA (N, N-diethyl phenylacetamide) based mosquito repellent cream Mosqshield®, applied at 1.0 mg/cm^2^ was taken as positive control for comparison with the test patch. Each trial was replicated at least three times on different days using three volunteers. The volunteers were assigned randomly for each trial. All the volunteers selected were non smokers, non alcoholic and had no known history of allergic reactions to mosquito bites. The trials were randomised between the investigators in order to minimise the bias. Written informed consent was obtained from all volunteers used in the present study. Number of mosquitoes attempted to settle for the blood feed were scored as landing, whereas those landed successfully and attempted to suck blood were scored as biting.

The percent repellency was calculated using equation–5

(5) $$\mathrm{Percent}\;\mathrm{repellency}\left(\mathrm{PR}\right)=C‒N\times 100/C$$

Where, C = Number of mosquitoes on control

N = Number of mosquitoes on test

The repellency index was calculated using the equation–6

(6) $$\mathrm{Repellency}\;\mathrm{index}\left(\mathrm{RI}\right)=\left(C‒N\times 100\right)/\left(C+N\right)\;$$

### Data analysis

The values obtained in the physiochemical characterisation were expressed as mean ± standard deviations. PR and RI obtained at different time intervals of the trials were compared using two-way ANOVA followed by Tukey-Krammer test of multiple comparisons at 95% confidence interval. Further, the PR and RI of deltamethrin loaded patch (test) and Mosqshield® cream (positive control) at different time intervals of test were compared using Student’s ‘t’ test.

The study project was approved by the Institutional Review Committee of Defence Research Laboratory, Tezpur and Ethical Review Committee of LGB Regional Institute of Mental Health, Tezpur. Informed written consents were obtained from the volunteers participated in the repellent trials.

## Results

### Physiochemical characteristics

The physicochemical characteristics of the deltamethrin-loaded mosquito repellent patches along with different combinations of EC and PVP have been presented in Table 1. The weight variation and the surface area of all the formulations varied from 0.46 ± 0.10 to 0.54 ± 0.06 and 13.64 ± 1.37 to 14.51 ± 0.68 respectively. The moisture uptake ranged from 3.42 ± 2.66 to 8.92 ± 5.79, while the moisture content was 2.60 ± 0.25 to 5.52 ± 0.43. The prepared patches were found to be uniform in thickness and variation in the thickness of all the formulations were in the range of 0.35 ± 0.01 to 0.57 ± 0.01. Thickness uniformity and low weight variation indicated the uniformity in the patches. The values of all the physicochemical parameters evaluated were in the appropriate range. The percent deltamethrin content was found to ranged from 50.35 to 77.60% among all the patches. The surface pH of the patches was ~6 which is similar to human skin pH, indicating that these patches were non-irritating to the skin. The kinetic study revealed that A5 bath formulation was best optimized and displayed Fickian diffusion controlled release mechanism governed by Higuchi kinetics (Table 1).

**Table 1:** Composition and physicochemical characterization of deltamethrin-loaded mosquito repellent patches

**Formulation batch**	**EC: PVP-K 30**	**Weight variation (gm)**	**Thickness (mm)**	**Surface area (cm**^**2**^**)**	**Moisture uptake (%)**	**Moisture content (%)**	**Deltamethrin content (%)**
A1	1:2	0.47 ± 0.07	0.42 ± 0.03	14.51 ± 0.68	7.31 ± 4.33	4.50 ± 0.60	50.35
A2	2:1	0.48 ± 0.06	0.41 ± 0.01	13.84 ± 0.66	4.67 ± 2.66	3.13 ± 0.10	75.76
A3	3:0	0.46 ± 0.10	0.51 ± 0.01	14.30 ± 1.41	3.42 ± 2.66	2.60 ± 0.25	77.60
A4	0:3	0.54 ± 0.06	0.57 ± 0.01	14.09 ± 1.68	8.92 ± 5.79	5.52 ± 0.43	62.05
A5	1.5:1.5	0.46 ± 0.04	0.35 ± 0.01	13.64 ± 1.37	6.23 ± 1.57	3.18 ± 0.11	66.41

### SEM analysis

**Figure 1:**
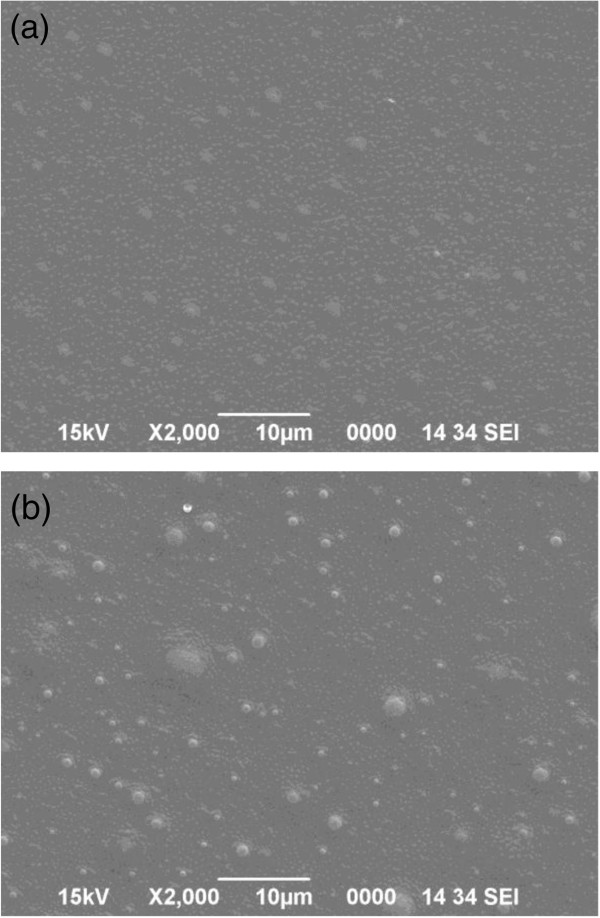
**SEM microphotograph.** Microphotograph of scanning electron microscope (SEM) showing the homogeneous dispersion of the deltamethrin in the polymeric matrices **(a)** blank patch **(b)** deltamethrin loaded patch (×2000).

SEM photographs demonstrate the homogeneous dispersion of deltamethrin in the polymeric matrices. The developed patches were spherical, smooth, less sticky, clear, homogenous and uniform as confirmed by SEM study (Figure 1a and b).

### FT-IR analysis

**Figure 2:**
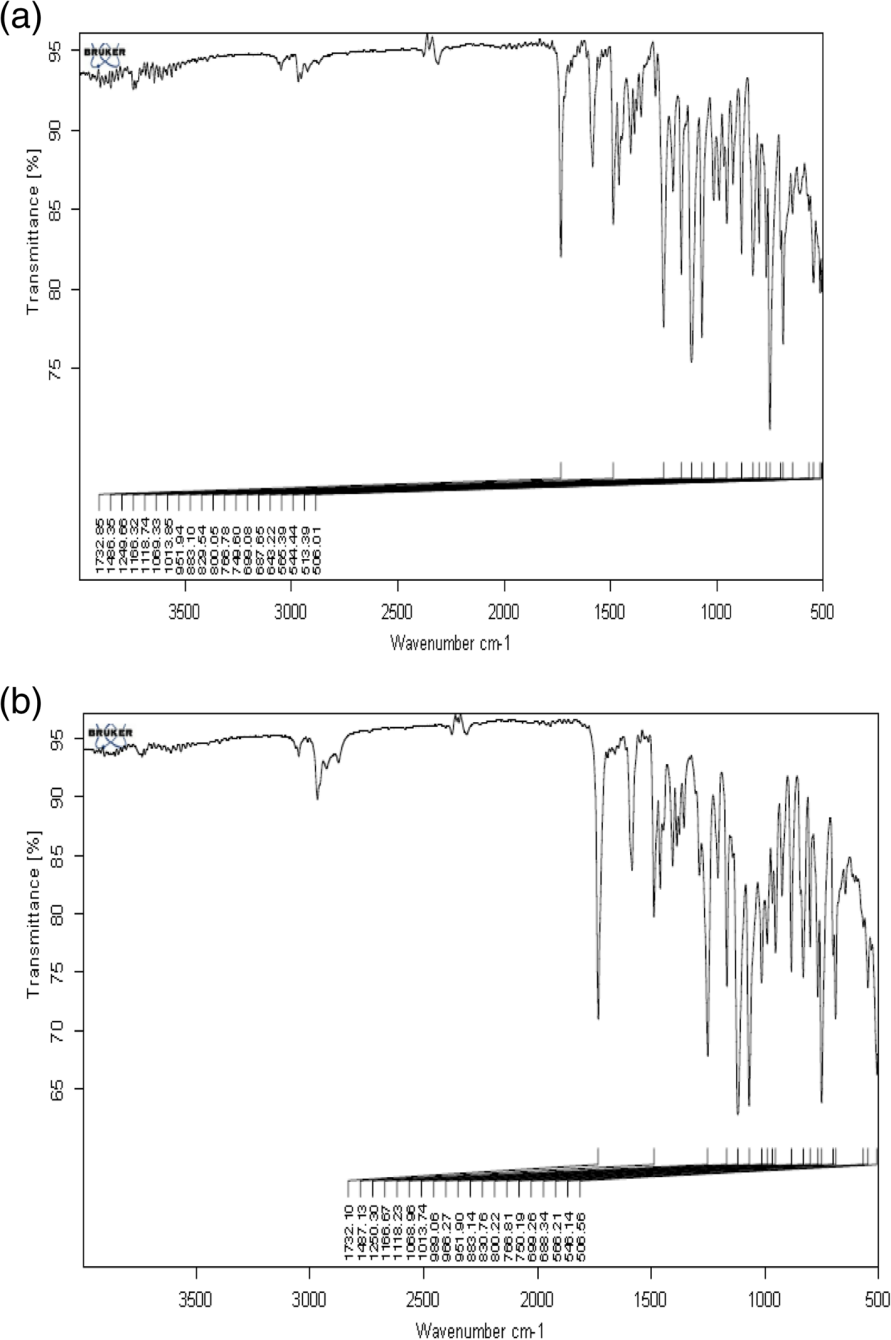
**FT-IR microphotograph.** Microphotograph of FT-IR spectroscopy **(a)** deltamethrin **(b)** deltamethrin loaded patch.

The FT-IR spectra of deltamethrin showed peaks, at 1732.85 cm^-1^ due to carbonyl compound, at 1486.35 cm^-1^ for ring stretch absorption C = C groups, at 1118.74.cm^-1^ due to aromatic C-O-C stretch, at 1013.85 cm^-1^ due to aromatic ethers of C-O band groups, at 749.60 cm^-1^ showed out of plane bending due to = C-H group and at 643.22 cm^-1^ due to strong stretch in aliphatic bromides (Figure 2a). In the deltamethrin loaded patch, the spectra of EC showed peaks at 3474.74 cm^-1^ due to O-H group, at 2973.06 cm^-1^ symmetric stretch due to the presence of C-H group, at 1481.98 CH_2_ bending and at 1374.66 cm^-1^ due to CH_3_ bending, while for PVP, the spectra showed peak at 3423.30 cm^-1^ due to amide N-H stretching, at 1644.92 cm^-1^ due to C = O group and at 1457.93 cm^-1^ due to methylene group bending absorption (Figure 2b). However, similar characteristic peaks related to deltamethrin were noticed with slight variations in deltamethrin loaded patches. Results suggested that the deltamethrin was stable in the patch formulation.

### DSC analysis

**Figure 3:**
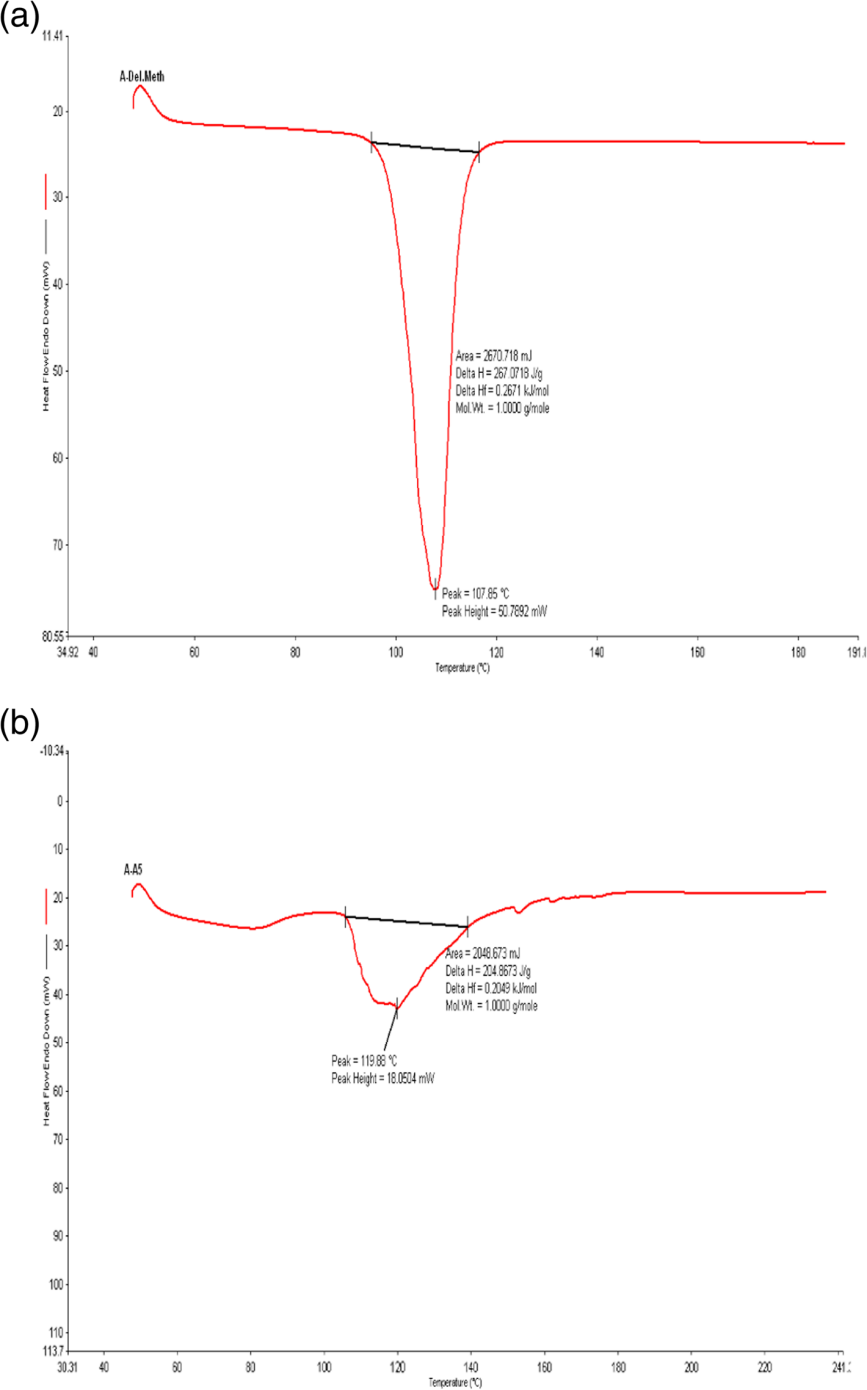
**DSC phase diagram.** Phase diagram of differential scanning calorimeter (DSC) **(a)** deltamethrin **(b)** deltamethrin loaded patch.

DSC analysis of deltamethrin showed a sharp endothermic peak at 107.85°C corresponding to its melting point (Figure 3a). Whereas, the deltamethrin loaded patch with EC: PVP showed a blunt endothermic peak at 119.88°C with slight change in melting point of deltamethrin towards higher temperature (Figure 3b). DSC results indicated that the formulation did not show any physical transformation and was stable.

### Repellency evaluation

**Figure 4:**
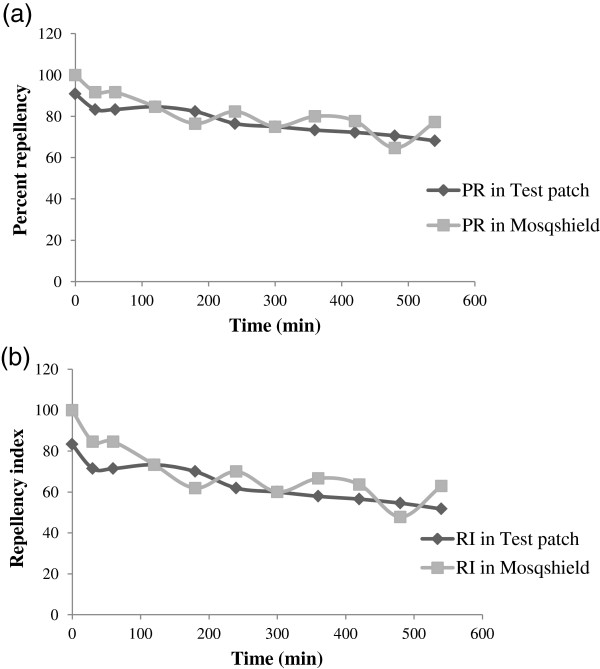
**Landing protection against *Ae. albopictus.*** Landing repellency of deltamethrin loaded patch and Mosqshield® against *Ae. albopictus***(a)** percent repellency **(b)** repellency index.

**Figure 5:**
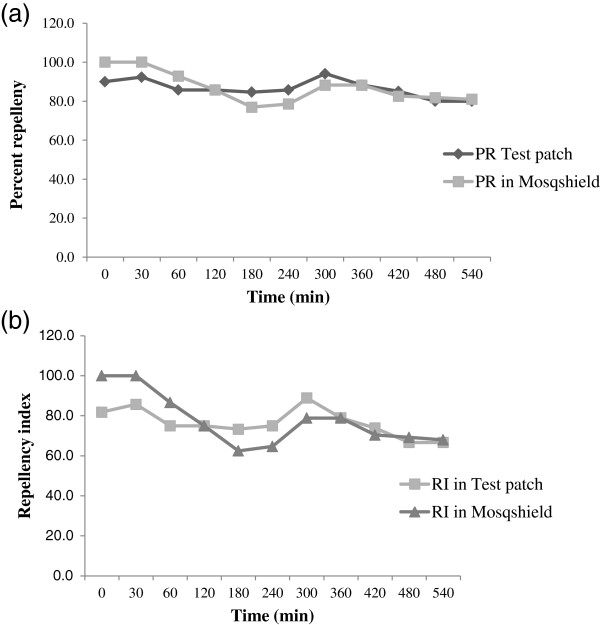
**Biting protection against *Ae. albopictus.*** Biting repellency of deltamethrin loaded patch and Mosqshield® against *Ae. albopictus***(a)** percent repellency **(b)** repellency index.

Repellent activity of the deltamethrin-loaded patches was evaluated up to 9 h and comparisons were made with DEPA based commercial mosquito repellent cream Mosqshield® under laboratory conditions. Randomly selected patches from all the batches did not show a significant difference in the repellent activity (Table 1). Test patches displayed a consistent landing repellency throughout the trials, as both PR (F = 1.64; p = 0.17) and RI (F = 1.69; p = 0.15) did not differ statistically at various time intervals. The comparison of the landing repellency of test patch and Mosqshield® cream has been shown in Figure 4a and b. At the beginning of the trial, the PR and RI of test patch were 90.9 and 83.3 respectively, while in Mosqshield® cream both PR and RI were 100 each. However, after 9 h of the application, the landing PR and RI of test patch decreased to 68.2 and 51.2, while in Mosqshield® it decreased to 77.3 and 62.9 respectively. No statistical difference was observed among the landing PR and RI of test patch and Mosqshield® cream at various time intervals (t ≥ 1.99; p ≥ 0.19). Biting repellency of test patches as compared to Mosqshield® has been depicted in Figure 5a and b. There was no significant difference among the biting PR (F = 0.34; p = 0.96) and RI (F = 0.37; p = 0.94) at various testing time intervals. The biting repellency of test patches and Mosqshield® was found to be statistically similar, as there was no difference among the PR (t ≥ 1.21; p ≥ 0.29) and RI (t ≥ 1.18; p ≥ 0.31) of test patch and Mosqshield® at various time intervals during the test.

## Discussion

Although the variety of mosquito repellents available now a days is remarkable, still the use of ULV insecticides remains a popular option for controlling the mosquitoes. In the present investigation, for the first time we showed that the use of ULV deltamethrin in a polymer patch is effective as repellent against laboratory reared *Ae. albopictus* mosquitoes. *Ae. aegypti* and *Ae. albopictus* are well known vectors responsible for the transmission of dengue fever in many countries [2,3,26]. The emergency interventions in dengue outbreaks rely on the indoor thermal fogging and outdoor ULV application of suitable insecticides [21,27]. While effective, these interventions are difficult to implement and sustain in the urban settings, where *Ae. aegypti* is more common and potential dengue vector [21,28]. Global climate change has led to the range expansion of mosquito species and resistance to the insecticides, therefore, limited exposure of insecticide is recommended in anti-mosquito efforts.

Some of the commercially available formulations may contain up to 40% synthetic insecticide and claim that the higher concentrations are most appropriate to use when, mosquito biting pressure and the risk of mosquito borne diseases is high and the environmental conditions promote rapid loss of repellent [29,30]. The deltamethrin patch formulation developed in the present study has minimal insecticide exposure for humans, thereby reducing the human-health and environment related risks. The moisture content (2.60 ± 0. 25 to 5.52 ± 0.43) of these patches was consistent with the increasing hydrophilic polymer (PVP) content, while the moisture uptake varied from 3.42 ± 2.66 to 8.92 ± 5.79. The variation occurred due to the presence of different ratios of polymers. High moisture uptake corresponds to the use of high concentrations of PVPK-30, which is hydrophilic and hygroscopic in nature. Low moisture uptake capacity has the advantage that it protects the patches from microbial contamination and bulkiness during high humid conditions. There was no difference in the length of the strips before and after cutting in longitudinal sections, indicating zero constriction and 100% flatness. Therefore, the patches maintained a smooth and uniform surface when applied on the human hand for evaluating the anti-mosquito efficacy. The FT-IR and DSC studies indicated compatibility between deltamethrin and the excipients used in the fabrication of the repellent patches.

Many of the synthetic repellent based products have not been proved to be efficacious in mosquito control [10]. However, some studies have suggested that synthetic insecticide based products are highly effective in repelling the mosquitoes and other hematophagous insects [8,16,30,31]. The synthetic insecticides have been found to be consistent in activity even after a long period of use, unlike the herbal based repellents, which provide repellency initially but after some time the activity ceases [7,9,16,32]. Other studies have indicated that many of the synthetic repellents while applied along with the other repellents provide more protection against mosquitoes as compared to applied singly [33]. The repellent patches developed and evaluated in the present study were assumed to provide protection against the mosquito up to 3-4 h only, but these patches were effective even after 8-9 h of the application and compared well with the commercial formulation Mosqshield®. These patches would have selective advantages over the cream and lotion based synthetic formulations. The cream and lotion based repellent formulations are comparatively less preferred because these are applied directly onto the bare skin and sometime causes discomfort to the user.

The deltamethrin loaded patches developed here were proven to be safe to the user and effective against mosquitoes. The patch is completely safe because the backing material used in the formulation acts as barrier for deltamethrin to penetrate into the human skin. The active component is slowly released into the surrounding atmosphere at a very low concentration. The patches were found to be stable and displayed very good physical properties at room temperature. The repellent patch formulation is a novel approach and provides alternate mechanism to deliver deltamethrin into the surrounding environment effectively. Present results could be helpful in developing specific uses such as providing mosquito repellents which would be useful during outdoor working hours.

## Conclusion

Deltamethrin loaded patches developed in the present study provided promising results in repelling the *Ae. albopictus* mosquitoes under laboratory conditions. The study emphasised that deltamethrin released to the environment at ultra low concentration could be a useful method for providing protection against mosquitoes in the tropical settings. However, the present findings relate to the application of the repellent patches against laboratory reared *Ae. albopictus* mosquitoes only, therefore, more detailed, multicentric and well replicated field studies are essential to establish long-term efficacy, practicability, affordability and acceptability of the developed patches against a variety of vector mosquitoes.

## Competing interests

The authors declare that they have no competing interests.

## Authors’ contributions

All the authors have contributed significantly to this study. PC, SD and VV conceptualized and designed the study. SB, KAD, AC and PC performed the analytical studies. SD and PC performed data analysis. BR, SD and KAD performed the repellency experiments. PC and SD prepared the manuscript. VV critically reviewed the manuscript. The opinions contained herein are the personal views of the authors only and are not to be considered as official views of the Department of Defence R&D, Govt of India. Mention of specific commercial products is for better understanding and does not constitute any recommendation. All authors read and approved the final manuscript.
